# Males and Females Gain Differentially from Sociality in a Promiscuous Fruit Bat *Cynopterus sphinx*

**DOI:** 10.1371/journal.pone.0122180

**Published:** 2015-03-20

**Authors:** Kritika M. Garg, Balaji Chattopadhyay, D. P. Swami Doss, A. K. Vinoth Kumar, Sripathi Kandula, Uma Ramakrishnan

**Affiliations:** 1 Ecology and Evolution, National Centre for Biological Sciences, TIFR, Bellary road, Bangalore 560065, India; 2 School of Biological Sciences, Madurai Kamaraj University, Madurai 625021, India; University of Freiburg, GERMANY

## Abstract

Sociality emerges when the benefits of group living outweigh its costs. While both males and females are capable of strong social ties, the evolutionary drivers for sociality and the benefits accrued maybe different for each sex. In this study, we investigate the differential reproductive success benefits of group membership that males and females might obtain in the promiscuous fruit bat *Cynopterus sphinx*. Individuals of this species live in flexible social groups called colonies. These colonies are labile and there is high turnover of individuals. However, colony males sire more offspring within the colony suggesting that being part of a colony may result in reproductive benefits for males. This also raises the possibility that long-term loyalty towards the colony may confer additional advantage in terms of higher reproductive success. We used ten seasons of genetic parentage data to estimate reproductive success and relatedness of individuals in the colony. We used recapture data to identify long and short-term residents in the colony as well as to obtain rates of recapture for males and females. Our results reveal that males have a significantly higher chance of becoming long-term residents (than females), and these long-term resident males gain twice the reproductive success compared to short-term resident males. We also observed that long-term resident females are related to each other and also achieve higher reproductive success than short-term resident females. In contrast, long-term resident males do not differ from short-term resident males in their levels of relatedness. Our results re-iterate the benefits of sociality even in species that are promiscuous and socially labile and possible benefits of maintaining a colony.

## Introduction

Animal social systems can be defined as diverse patterns of social interactions and the resulting relationships among the members of a society [1] or as a synthesis of the nature, quality, and patterning of the relationships among the members of a population [2]. While sociality in animals is interesting to observe, it is a byproduct of individual strategies to optimize fitness [3]. Individuals decide to join or form groups, when the benefits of group living outweigh the costs [4, 5]. However, all individuals within a group do not gain similar benefits [6]. Individual benefits depend on the size of the group, sex, age and social status of the individual [7]. Benefits (measured in reproductive success) can also depend upon the time an individual spends at the group. In barnacle geese, the duration of the pair bond determines the lifetime reproductive success. Male-female pairs, which form long-term pair bonds, have a higher lifetime reproductive success than pairs forming short-term bonds [8]. Similarly, non-territorial males of the greater sac-winged bat occupy harem-male territories in hierarchal fashion, where the hierarchy is determined by the time spent by males around the colony. Harem males obtain most of the reproductive benefits in this species [9].

Certain benefits of sociality are also sex specific as males and females experience different evolutionary constraints. While distributions of females and their interactions are dependent on predation risks and resources, males segregate and interact in accordance with reproductive opportunities [1, 10]. For example, in Assamese macaques, males derive social dominance gains from coalitions, which enhance their reproductive success [11]. Similarly, among feral horses, females form long-term bonds, which increase foal birth rate and survival, thus affecting reproductive success of the females [12]. The investigation of sex-specific strategies is specifically important to understand the benefits of group membership in mixed sex societies [11].

Estimates of reproductive success and kinship are commonly used to quantify the benefits of social associations, and together they form the basic indicators of mating and social dynamics [2]. In many cooperatively and non-cooperatively breeding societies, kinship plays an important role in social organization and breeding [13, 14]. While kinship quantifies genetic relatedness between individuals and may not directly indicate fitness, reproductive success in contrast can directly quantify fitness consequences of individual-based strategies. In other words, an understanding of the interplay between the genetic nature of social relationships (kinship) and reproductive benefits can provide deeper insight regarding the evolution and maintenance of groups [15]. For example, in baboons, females form strong social ties with relatives. These bonds affect their possibility of finding coalitionary support, and consequently enhance their social rank and reproductive success [16]. In contrast, in male chimpanzees [15, 16], male Assamese macaques [11] and female feral horses [12] the coalitions are maintained between unrelated individuals. In all of the above systems however, social bonds are strong and can be maintained throughout the lifespan of some individuals. While such systems tend to be well studied behaviorally, we know less about flexible, labile societies, in part because behavioral observations in such systems are difficult.

In this study, we made an attempt to understand individual strategies and the benefits of group living in a flexible social system with high turnover of individuals. We studied group living in the promiscuous harem-forming fruit bat, *Cynopterus sphinx*. Males in this species construct tents and females join them. An association of a resident male with one or more females is commonly called a ‘harem’. A colony consists of multiple harems along with solitary males clustered in an area [17]. However, these harems are labile and maintained throughout the year despite the presence of well-defined mating periods [18]. There are two periods of sexual activity in this species (February to March: dry season and October to November: wet season; [19, 20]) and parturition occurs between February to March, dry season and again between June to July, wet season [20]. Reports suggest total natal dispersal of the weaned juveniles [17, 20, 21]. A high turnover rate of individuals at the colony is observed across seasons, with nearly 65% of the individuals in each season being immigrants [21]. Females move between harems and colonies, and males have no control over female movement [17, 18, 21]. Males do not obtain any reproductive benefits from maintaining harems [21], but the roost seems to be important for social organization [18].

We investigated the benefits of flexible group living and the possible reasons behind the maintenance of colonies in *C*. *sphinx*. We also investigated if males and females adopt different strategies and gain differential benefits of group living. We did so by performing behavioral and genetic analyses on a five-year (ten seasons) dataset consisting of tagged individuals from the colony. Considering the lability of the individuals, we first divided our data set into two categories: individuals who were captured at the colony at least twice (i.e., they have a propensity to stay at the colony and are likely to be loyal to the colony) were called ‘long-term residents’ and individuals who were captured at the colony only once were called ‘short-term residents’ (these individuals were never again captured at the colony and were most likely visitors at the colony). We specifically tested a) if long-term residents have a higher reproductive success than short-term residents, b) if there are differences between males and females in long-term and short-term resident strategies, c) if the colony is formed by relatives and d) if the proportion of related males and females differs in a colony. For our study species we predict that the long-term residents may obtain higher reproductive benefits within the colony, when compared to the short-term residents as they have direct access to reproductively active individuals within the colony. Further, males and females might differ in their resident strategies, as males’ investments in maintaining a harem and colony are higher. Males construct the tents and spend less time foraging than females to protect the tents [17, 18, 21]. They also display significantly higher roost fidelity than females [17]. Further relatedness may play an important role in maintaining harems within the colony. Previous work on social organization in *C*. *sphinx* suggested that males and females within a harem were related [22].

## Methods

### Ethics statement

This study and the sampling were approved by the institutional ethics committees (Internal Research Review Board (IRB), Ethical Clearance (EC), Biosafety and Animal Welfare committee approval to BC dated 21–11–2005 Madurai Kamaraj University and Institutional Animal Ethics Committee (IACE) to UR id UR-3/2009, National Centre for Biological Sciences). All sampling sites are located on private property (GPS coordinates: CSA colony, 8.638^0^ N, 77.958^0^ E; CST colony, 8.631^0^ N, 77.904^0^ E and SCH colony, 8.631^0^ N, 77.899^0^ E). We obtained permission from owners for our work. This species is classified under Least Concern category in the IUCN red list. Besides, fruit bats are considered as vermin under the Indian Wildlife protection Act (1972) and hence no permits are required to sample them. KMG, BC, DPS and SK performed nonlethal tissue sampling for molecular genetic analyses. 4mm biopsy punch was obtained from wing membrane (area where no major blood vessels were present) of each bat and stored in 95% ethanol.

### Field sampling

We studied the importance of group living in a *C*. *sphinx* colony at Samyathu village (8.638^0^ N, 77.958^0^ E) in Tamil Nadu, India. This colony is a part of long-term study on mating system and social system of *C*. *sphinx*. Data collected for ten consecutive seasons from 2008 to 2012 were used for all analyses. Additionally two colonies located near the main colony (CST: 8.631^0^ N, 77.904^0^ E and SCH: 8.631^0^ N, 77.899^0^ E) were also sampled for two seasons in 2012. Details of sampling are reported in Garg et al., [21]. In short, we used hoop nets with extendable aluminum poles to capture bats at the roosts. We captured bats approximately four weeks after parturition. By this time most females had given birth and the pups were old enough to sample but had not yet weaned. We captured the entire colony within two days. We performed a visual census to determine colony size before sampling. Overall, we obtained data from five wet and five dry seasons. Individuals roosting near the colony were also captured and sampled. These additional samples helped us in the assignment of paternity with more confidence.

### Microsatellite Genotyping

Methods for DNA extraction and microsatellite typing follow Garg et al., [21]. We used Ampli-Taq Gold (Applied Biosystems) for PCR amplification for samples from seasons one to six and PCR Multiplex mastermix (MM; Qiagen) for the rest of the samples. We scored all microsatellite alleles twice with GENEMAPPER version 4.0 (Applied Biosystems) and standardized the entire dataset using TANDEM [23]. We determined the error rate for microsatellite typing by regenotyping 5% of samples from seasons one to six (for Ampli-Taq gold) and seasons seven to ten (for MM) respectively. We also regenotyped 5% of samples from season one to six with MM to test for consistency between Ampli-Taq Gold and MM. Overall 12% of the samples were regenotyped.

### Parentage assessment

Details of parentage assignment are described elsewhere [21]. In brief, we used Cervus 3.0 to assign paternities to the pups born over the five-year period [24, 25]. Cervus uses a likelihood-based method for assigning genetic relationships [24]. We assigned the mothers season-wise, allowing a 1% error rate and assuming a 90% capture probability. We performed 10,000 simulations to estimate delta values for assignment at 80% and 95% confidence. For paternity analysis we only considered pups whose mother could be assigned at 80% confidence (or higher). For seasons one to four, we considered all adult males (colony and extra colony males) captured over the entire five-year study period for paternity analysis. For seasons five and six, we also considered males born in season one and two as candidate fathers as they were sexually mature during the mating periods for season five and six. Similarly for seasons seven and eight males born in season one to four and for seasons nine and ten males born in season one to six were also considered as candidate fathers alongside all adult males. For paternity analysis, we carried out 10,000 simulations assuming 70% capture probability and allowing for 1% error rate.

### Recapture data

We tagged individuals with unique color-coded beads (0–9). We tagged only males during season one and two whereas all individuals captured during seasons three to ten were tagged. We used four beads to tag adults and three beads for juveniles. The tags weighed about 0.45g. We threaded the beds together into a necklace and crimped the two ends using a copper wire spring. We cut the excess wire to avoid any inconvenience to the animals. To avoid any injury to the bat we ensured that once wound, the necklace could move freely around the bat’s neck. These tags are known to last at least ten years and are not known to cause any harm to the animals (SK personal communication). We specifically used these tags to identify recaptures. Additionally, we also used genetic data from microsatellite genotyping to identify genetically recaptured individuals from seasons one and two. We used Cervus 3.0 to identify genetic recapture. We allowed upto one mismatch to identify recaptures. We determined the accuracy of genetic recapture by comparing field data with genetically resampled individuals (n = 52). We further tested for significant difference between recapture rates of males and females. We performed paired t-test on proportion of males and females recaptured at the colony in each season to test if there was any significant difference in recapture rates.

We also conducted demographic analyses using mark-recapture methods in MARK program [26]. We analyzed the capture history data using the Schwarz and Arnason [27] parameterisation of the Jolly-Seber capture recapture modelling approach. In this approach, parameters estimated include capture probability at occasion *i* (*p*_*i*_), survival probability from occasion *i* to *i+1 (ϕ*_*i*_), the super-population of individuals that are ever present in the sampled colony (*N*) and the probability that an individual from the super-population enters the sampled colony between *i* and *i+1* (*b*_*i*_). Number of net new entrants (*B*_*i*_) and size of the colony size at each sampling occasion *(N*_*i*_) are estimated as derived parameters. For our dataset *p*_*i*,_ *ϕ*_*i*_and *b*_*i*_ were modelled either as constant, as varying across time, varying by sex or varying by season. Combinations of these covariates were modelled as having either additive effects or as interacting with each other. *N* was always estimated separately for each sex. We used Akaike’s Information Criterion corrected for sample size AIC_C_ [28] to select the best model to describe our data.

### Relatedness estimation

We used the program Coancestry [29] to estimate genetic relatedness between individuals. As relatedness estimates are dependent on the population structure and the quality of genetic data, we first performed simulations in Coancestry to determine the best relatedness estimator for our study system. We simulated 100 dyads for each of the following relationships: full-sibs, half-sibs, first, second cousins and unrelated; and allowed for 1% error in genotyping. To determine the best estimate, we further calculated the correlation between the true value and the seven estimates of relatedness implemented in Coancestry.

### Long-term residents v/s short-term residents

We concatenated the data across all ten seasons and tested if long-term residents have higher reproductive success than short-term residents. We performed Wilcoxon rank sum test to investigate significant differences between long-term and short-term residents, as the data on reproductive success was not normally distributed. We also performed a correlation between number of recaptures and the reproductive success of individuals. To control for recapture rate, we normalized the reproductive success of the long-term residents by the number of recaptures and again compared the normalized reproductive success of long-term residents to short-term residents.

We also tested if long-term residents were more related compared to all individuals captured at the colony. We used a bootstrap approach to compare the relatedness of recaptured individuals to all individuals. We tested if the probability of obtaining a mean relatedness value from randomly selected individuals from the colony was greater than mean relatedness of long-term residents. We also repeated the analysis separately for males and females to test if there was any sex-specific pattern. All statistical tests were performed in R 2.15.3 [30].

## Results

We captured a total of 635 adults across ten seasons including recaptures (Table A in S1 File). In addition, 177 adults were captured around the main colony of interest (distance varied form 0.27 km to 6.54 km). Capture success varied from 70% to 95% across seasons (Table B in S1 File). Of the initial ten loci, we could use only eight loci for paternity analyses (see [21]). We genotyped 762 individuals. The average polymorphic information content (PIC) for these markers was 0.7, and the error for assigning first parent based on allele frequency analysis was 1 in 50 while error in assigning the second parent, knowing the first was 1 in 1000. The number of alleles, heterozygosity and PIC for each locus is listed in Table C in S1 File. The overall error rate estimated from regenotyping was 0.005 (Table D in S1 File). While testing the accuracy of the genetic data for recaptures, we observed that out of 52 samples, two samples showed disagreement between genetic assignment and tag-based assignment. We discarded these two individuals from further analyses. We could assign mothers to 91% of the pups and subsequently fathers could be assigned to 181 of these pups (71%). We recaptured 41 males and 69 females at least once during the study period (Table 1). Overall the recapture rates increased with time (Table 2). Males had a significantly higher tendency to be recaptured in the colony than females (paired t-test, p value = 0.035, Tables 1 and 2).

**Table 1:** Total number of adult long-term and short-term residents at the colony.

	Long-term residents	Short-term residents
Males	41	72
Females	69	108

**Table 2:** Season-wise proportion of individuals recaptured.

Season	No. of individuals recaptured		Proportion of individuals recaptured	
	Male	Female	Male	Female
S2	6	10	0.43	0.36
S3	6	11	0.60	0.37
S4	11	15	0.61	0.56
S5	6	21	0.60	0.49
S6	6	13	0.46	0.62
S7	7	12	0.78	0.44
S8	9	12	0.82	0.57
S9	6	10	0.75	0.56
S10	8	12	0.62	0.55

We observed that based on the AICc criterion the best model for demographic analyses was the one where survival probability varied over time, capture probability was constant and probability of entry was influenced by an interaction of sex and time {*phi(t)*,*p(*.),pent(s*t),*N(s)*}, with ΔAIC values of other models being >10. In this model, estimated capture probability (mean (SE)) was 0.366 (0.027) and did not differ between males and females. Survival probability ranged from 0.654 to 1 depending on the sampling occasion. The male super-population was smaller (43.69 (2.81)) than that of the females (76.9 (3.95)). The probability of the entry of both males and females into the colony was zero in the wet season, but not in the dry season, suggesting that recruitment into the study population occurred primarily in the intervals spanning the wet season. The model did not find a consistent trend in male versus female entry probabilities.

**Fig 1:**
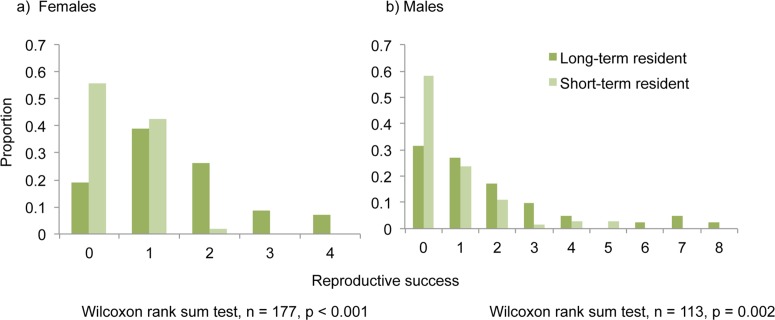
Distribution of reproductive success of long-term and short-term resident a) females and b) males.

**Fig 2:**
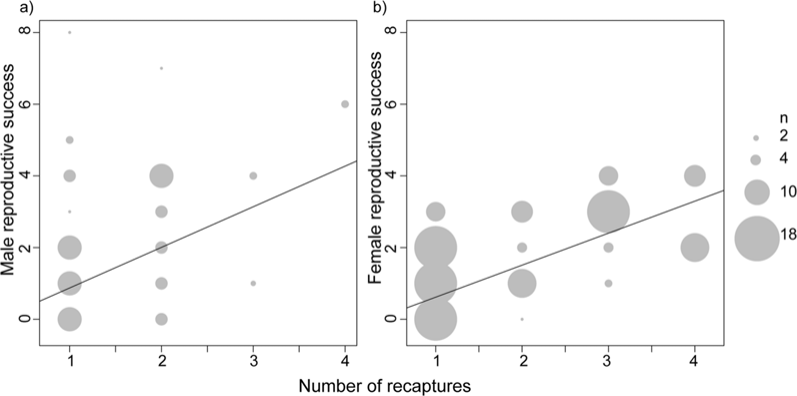
Correlation between recapture rate and over all reproductive success for a) females and b) males.

The overall reproductive success of long-term residents (males and females) was significantly higher than short-term residents (p value males = 0.002; p value females < 0.001, Fig. 1). The reproductive success of long-term resident males was twice than that of short-term residents (mean reproductive success of long-term resident males = 1.8, mean reproductive success of short-term residents = 0.75). This effect was magnified for females, and long-term resident females (mean reproductive success = 1.46) were three times more successful than short-term resident females (mean reproductive success = 0.46). The correlation between the number of times an individual was recaptured and the overall reproductive success was highly significant for females (p value < 0.001, adjusted R^2^ = 0.35) and was also significant for males, though with very low R^2^ (p value = 0.033, adjusted R^2^ = 0.09, Fig. 2). The normalized reproductive success of the long-term residents was still significantly higher than that of the short-term residents (p value males = 0.03; p value females < 0.001).

**Fig 3:**
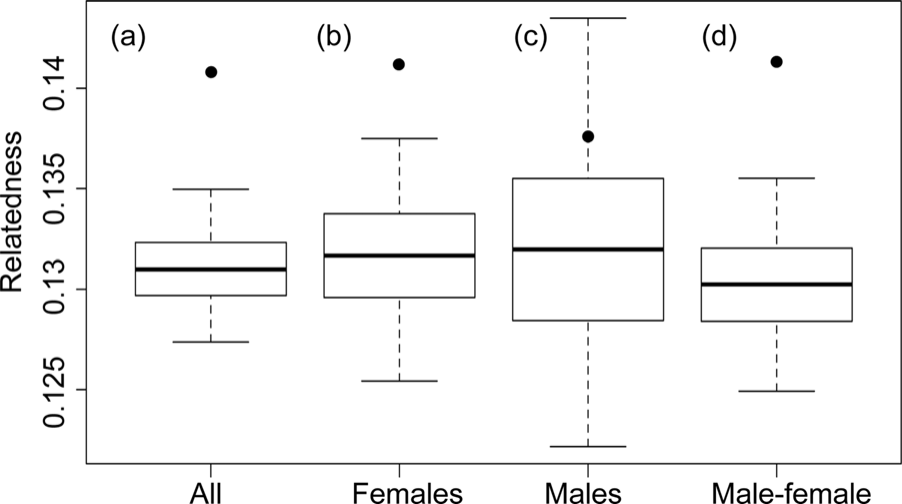
Modified boxplots depicting the simulated and observed relatedness of recaptured a) adults, b) females, c) males and d) males-females. The box depicts the 25^th^ and 75^th^ quartiles, the solid line is the median and the whiskers are the 2.5% and 97.5% percentiles.

Based on the simulations we observed that the dyadic ML relatedness estimates had the highest correlation with the true value, followed by the Trio ML estimates (Table E in S1 File). Hence we used dyadic ML estimates for all the analysis. We observed that the adults in the colony were positively related (Fig. 3). Further, the overall relatedness of the long-term residents was significantly higher than the average relatedness of all individuals in the colony (p value < 0.001; Fig. 3a; Table 3). Long-term resident females were significantly related compared to colony females (p value = 0.001; Fig. 3b; Table 3), whereas relatedness of long-term resident males from other males in the colony did not differ significantly (p value = 0.22; Fig. 3c; Table 3). We also observed that long-term resident males and females were more related than any random male-female pair (p value < 0.001; Fig. 3d; Table 2).

**Table 3:** Bootstrap test to investigate the relatedness of recaptured individuals to the colony.

Sl. No.	X	Number of pairs in X	Y	Number of pairs in Y	p value
1	Recaptured individuals	6,216	All individuals	45,753	< 0.001
2	Recaptured females	2,485	All females	18,721	0.003
3	Recaptured males	820	All males	5,886	0.16
4.	Recaptured males and females	2,911	Male and female pairs	21,146	< 0.001

## Discussion

We examined the evolutionary benefits of group membership for males and females using long-term genetic and behavioral data in a flexible and gregarious society of fruit bats. Individuals at the colony were tagged, and data on recapture was used to differentiate between long-term and short-term residents at the colony. This framework allowed us to differentiate between the benefits that males and females might obtain for maintaining long-term bonds and being a stable part of the colony.

### Difference in male and female residence strategies

We recaptured approximately 57% of the individuals at the colony. Similar to prior reports on male fidelity [17, 21], males in this colony remain significantly more loyal to the colony than females (Tables 1 and 2). In terms of the benefits accrued from a prolonged association with the colony, our observations reveal that while males in our study colony only gain reproductive benefits (Fig. 1b), females gain both reproductive benefits as well as possible kin-benefits (Figs. 1a and 3b). Because females possibly gain more benefits than males, it can be expected that they should be more loyal to the colony and therefore show a higher rate of recapture. However, we observe quite the opposite. One possible reason for this disconnect may be the fact that most females are assured of reproductive success each season; whereas approximately 50% of the males are unsuccessful (based on five year parentage data). Thus from the perspective of males, merely the possibility of reproductive benefit might promote higher chance of being a long-term resident within the colony. Further, males’ invest more in constructing and protecting tents from other males. Their investment in maintaining harem and colony is higher than females [17].

The mark-recapture based models imply that there is no differences in capture probability between males and females, suggesting the observed difference in the recapture rates are unbiased. Unfortunately, apart from capture probability, the demographic parameters estimates and the predictions made based on reproductive success estimates are not comparable. Future studies where sampling is explicitly designed to link these types of data might yield more comprehensive insights.

### Long-term residents gain more reproductive benefits

Both long-term resident males and females in our colony gained direct benefits of group living in terms of higher reproductive success compared to short-term residents (Fig. 1). The reproductive success normalized for recapture rate was still greater than the reproductive success of short-term residents, indicating that colony fidelity might be an effective strategy that guarantees successful mating opportunities at least within the colony, reiterating our previous observations [21]. Similar results were observed in lesser flat-headed bats where resident males higher reproductive success compared to solitary or bachelor males [31]. Females in this species form matrilines in bamboo stems and males may remain associated with females (residents) or form bachelor groups or remain solitary. Solitary or bachelor group males obtain only few paternities within the social unit compared to resident males, suggesting the importance of maintaining social bonds and site fidelity [31].

It is interesting to note the difference in correlation strength between sexes for reproductive success as a function of recapture rate (Fig. 2). Prolonged stay at the colony may not provide additional benefits to males’ but assures higher reproductive success to females. Most females in *C*. *sphinx* reproduce and within season variance in female reproductive success is low [17, 21]. This implies that being a stable part of the colony provides females with a stable space to access males and successfully breed and these colonies might provide the females with opportunity for mate choice. However these results should be interpreted with caution as recapture rate might contribute in inflating the results (Fig. 2a). Actual information on time spent in the colony (for example, obtaining recapture data using PIT tags) and correlation with reproductive success might provide a clearer picture on relationship between duration of colony membership and reproductive success.

We recognize that, while individuals can be unambiguously categorized as long-term residents if captured twice or more, individuals captured once may include a mixture of true short-term residents (by definition, these cannot be captured again) and some unknown proportion of long-term residents (present for two or more seasons but did not happen to get recaptured). However, it has to be noted that the effect of such a mixture within the group categorized as short-term residents would make our estimated difference in reproductive success a conservative one (both in terms of a smaller estimated difference as well as a larger P-value associated with the test statistic), as having such a mixture would bring the mean value of the 'short-term' resident group closer to the long-term resident mean.

### Long-term residents gain indirect kin benefits

Our observations suggest that relatedness between long-term residents was significantly higher than average colony relatedness (average relatedness of individuals at the colony varied from 0.12 to 0.15 across seasons (Table E in S1 File)), indicating that the social system of *C*. *sphinx* might be kin-based (Fig. 3). It seems that within our study colony relatedness may play an important in the structuring of the social organization, especially for females. Although the relatedness among long-term resident females is not very high (mean = 0.14; Fig. 3b), it was significantly different from randomly picked females. In many primates, cetaceans and carnivores, relatedness is an important factor to maintain long-term associations, especially for females [32]. Related females obtain both inclusive fitness benefits and benefits from maintaining such long-term coalitions [16]. Female baboons are known to invest heavily in maintaining these bonds even during poor resource conditions, indicating the importance of these bonds [16]. Similarly in many bats (*Rhinolophus ferrumequinum*, *Myotis bechsteinii*, *Plecotus auritus*, *Myotis septentrionalis*) related females group to form nursery colonies [33]. These bonds may help females during pregnancy and lactation [33, 34]. Presence of related females within our colony may provide the raw materials for possible kin selection. Presence of related females in the colony is in contrast to earlier short-term studies on *C*. *sphinx* [22, 35].

Interestingly, relatedness could not explain male loyalty to the colony (Fig. 3c, Table 2) whereas reproductive benefits could. It is possible that relatedness does not play an important role in social organization for males of *C*. *sphinx*, as they do not form coalitions to defend the roost like in other harem forming bats (for example, in *Artibeus jamaicensis*, related males defend larger harems and share paternity and such coalition effectively reduce the chances of extra-harem paternity, [36]) and other mammals (for example Lions [37] and Chimpanzee [15, 38]). It is possible that unrelated males provide females with more options for mate choice [22]. However, we cannot rule out indirect kin benefits that males may obtain by being related to females (Fig. 3d). Similar to Chattopadhyay et al., [22] we observed significant relatedness between long-term resident males and females (Fig. 3d), emphasizing the importance of kinship in social organization in *C*. *sphinx*. Similar relatedness patterns were also observed in Indo-Pacific bottlenose dolphins [39], wherein females were related to other females and males within a group but males were unrelated to each other. The authors hypothesized that reduced risk of infanticide and harassment, group defense and cooperative foraging might be the possible reasons for such relatedness patterns. While some or all these possibilities might as well be true to our study system, further long term studies incorporating extensive behavioral observations alongside genetic and demographic data may address these questions to a greater detail.

## Conclusion

Based on our study we conclude that the colony is the main social unit in *C*. *sphinx*. By maintaining colonies individuals obtain reproductive benefits and possible kin benefits. Overall, our study highlights the importance of differentiating the benefits of sociality obtained by each sex. Males obtain direct reproductive benefits while females appear to obtain both direct reproductive and possible indirect kin benefits of group living. We also observe difference in strategies employed by males and females. Significantly higher proportion of males stay back in the colony compared to females indicating a potentially higher reward of ‘long-term resident’ strategy for males. Further work is required to determine the benefits obtained by short-term residents at the colony.

## Supporting Information

S1 File Supporting tables. Table A, Number of individuals captured in each season. Table B, Season-wise capture success. Table C, Summary statistics of the microsatellite markers used in this study. Table D, Genotyping error rate. Table E, Correlation between true relatedness values and various methods to estimate relatedness. Table F, Average relatedness between adults in the colony.
(DOC)

S2 File Details on the age, sex, and location of captured individuals along with the microsatellite genotypes used for the parentage analysis. (XLS)
