# Supplementary material for: Males and Females Gain Differentially from Sociality in a Promiscuous Fruit Bat Cynopterus sphinx
Source: PLoS One. 2015 Mar 20;10(3):e0122180. doi: 10.1371/journal.pone.0122180 (PMC4368723; doi:10.1371/journal.pone.0122180)
Supplement: S1 File — Table A, Number of individuals captured in each season. Table B, Season-wise capture success. Table C, Summary statistics of the microsatellite markers used in this study. Table D, Genotyping error rate. Table E, Correlation between true relatedness values and various methods to estimate relatedness. Table F, Average relatedness between adults in the colony. (DOC) [file pone.0122180.s001.doc]

**SUPPORTING INFORMATION**

**TABLES**

Table A: Number of individuals captured in each season.

| Season | Adults (Males/ Females) | Offspring |
| --- | --- | --- |
| S1 (main colony) | 74 (22/ 52) | 47 |
| S2 (main colony) | 42 (14/ 28) | 22 |
| S3 (main colony) | 40 (10/ 30) | 23 |
| S4 (main colony) | 39 (18/ 27) | 19 |
| S5 (main colony) | 53 (10/ 43) | 33 |
| S6 (main colony) | 34 (13/ 21) | 10 |
| S7 (main colony) | 36 (9/ 27) | 23 |
| S8 (main colony) | 32 (11/ 21) | 16 |
| S9 (main colony) | 26 (5/ 21) | 18 |
| S10 (main colony) | 34 (12/ 22) | 14 |
| Periphery | 177 (76/101) | 55 |

Table B: Season-wise capture success. For season 6 the census data sheet was misplace and hence we do not have information on the capture success for this season.

| Season | Total number of adults captured | Number of individuals escaped | Capture success (in %) |
| --- | --- | --- | --- |
| S1 | 74 | 10 | 88.10 |
| S2 | 42 | 2 | 95.46 |
| S3 | 40 | 10 | 80.00 |
| S4 | 39 | 8 | 82.98 |
| S5 | 53 | 17 | 75.71 |
| S6 | 34 | - | - |
| S7 | 36 | 7 | 83.72 |
| S8 | 32 | 13 | 71.11 |
| S9 | 26 | 11 | 70.27 |
| S10 | 34 | 5 | 87.18 |

Table C: Summary statistics of the microsatellite markers used in this study.

| Locus | Number of alleles | H_Obs_ | H_Exp_ | PIC |
| --- | --- | --- | --- | --- |
| CSP1 | 12 | 0.90 | 0.83 | 0.81 |
| CSP2 | 7 | 0.75 | 0.75 | 0.71 |
| CSP3 | 9 | 0.45 | 0.51 | 0.46 |
| CSP4 | 7 | 0.74 | 0.71 | 0.67 |
| CSP5 | 17 | 0.86 | 0.86 | 0.84 |
| CSP6 | 12 | 0.86 | 0.87 | 0.85 |
| CSP7 | 13 | 0.60 | 0.78 | 0.75 |
| CSP9 | 7 | 0.58 | 0.57 | 0.52 |

Table D: Genotyping error rate

| Comparison | Error rate |
| --- | --- |
| Ampli-Taq Gold | 0.004 |
| PCR Multiplex master mix | 0 |
| Ampli-Taq Gold v/s PCR Multiplex master mix | 0.01 |

Table E: Correlation between true relatedness values and various methods to estimate relatedness.

| Relatedness estimate | Correlation to true value |
| --- | --- |
| Trio ML | 0.689 |
| Wang | 0.654 |
| Lynch Li | 0.629 |
| Lynch Rd | 0.633 |
| Ritland | 0.383 |
| Queller Goodnight | 0.648 |
| Dyad ML | 0.691 |

Table F: Average relatedness between adults in the colony.

| Season | Average relatedness | Standard deviation |
| --- | --- | --- |
| S1 | 0.13 | 0.15 |
| S2 | 0.14 | 0.15 |
| S3 | 0.13 | 0.15 |
| S4 | 0.14 | 0.15 |
| S5 | 0.15 | 0.16 |
| S6 | 0.15 | 0.16 |
| S7 | 0.15 | 0.17 |
| S8 | 0.14 | 0.15 |
| S9 | 0.13 | 0.14 |
| S10 | 0.12 | 0.14 |
